# Cost-Effectiveness of a New Nordic Diet as a Strategy for Health Promotion

**DOI:** 10.3390/ijerph120707370

**Published:** 2015-06-30

**Authors:** Jørgen Dejgård Jensen, Henrik Saxe, Sigrid Denver

**Affiliations:** 1 Department of Food and Resource Economics, University of Copenhagen, Rolighedsvej 25, 1958 Frederiksberg C, Denmark; E-Mail: sd@ifro.ku.dk; 2 DTU Management Engineering, Global Decision Support Initiative, Danish Technical University, Produktionstorvet, Building 424, 2800 Kgs. Lyngby, Denmark; E-Mail: henrsa@dtu.dk

**Keywords:** New Nordic Diet, environmental impact, public health, cost-effectiveness

## Abstract

Inappropriate diets constitute an important health risk and an increasing environmental burden. Healthy regional diets may contribute to meeting this dual challenge. A palatable, healthy and sustainable New Nordic diet (NND) based on organic products from the Nordic region has been developed. This study assesses whether a large-scale introduction of NND is a cost-effective health promotion strategy by combining an economic model for estimating the utility-maximizing composition of NND, a life cycle assessment model to assess environmental effects of the dietary change, and a health impact model to assess impacts on the disease burden. Consumer expenditure for food and beverages in the NND is about 16% higher than currently, with the largest relative difference in low-income households. Environmental loads from food consumption are 15%–25% lower, and more than 18,000 disability-adjusted life years (DALY) will be saved per year in Denmark. NND exhibits a cost-effectiveness ratio of about €73,000–94,000 per DALY saved. This cost-effectiveness improves considerably, if the NND’s emphasis on organic and Nordic-origin products is relaxed.

## 1. Introduction

Inappropriate diets low in fruits, vegetables, whole grain products and seafood omega-3 fatty acids and high in meat, sugar and saturated fats constitute an important risk factor for several diseases, and hence for mortality, morbidity, quality of life and societal costs [1]. Increasing globalisation of food sourcing is furthermore considered to constitute a growing environmental burden. One proposed solution to this dual challenge for society is the development of healthy, regional diets based on local products, such as e.g., the Mediterranean diet [2]. Inspired by recent years’ success of the “New Nordic Cuisine” in gourmet restaurants [3], a New Nordic Diet (NND) has been developed based on products from the Nordic region, with a high score in palatability, healthiness and sustainability. Consumer interest is expected to be driven by a clear Nordic gastronomic identity, which could make the diet appealing and “trendy”. The principles of the New Nordic Diet have been delineated in Mithril *et al.* [4] (supplement Table S1), and are described by the following overall ambitions and guidelines:

- Gastronomic potential and Nordic identity—dishes based on high-quality organic food products with a Nordic origin and cultural heritage. Tastes from arctic seafood, and colour and flavor variation from plant foods, such as berries, cabbages, roots, legumes, potatoes and herbs should contribute to creating a Nordic identity.
- Health—relatively low meat intake and high intake of legumes, vegetables, fruit, whole grains, seafood, potatoes, nuts, herbs, *etc.*, compared with the average diet in many Western countries, including Denmark. This dietary composition should contribute to the prevention of health disorders such as weight gain, type 2 diabetes, cardiovascular diseases and cancer, but should also help maintaining and improving general physical, mental and social well-being via.
- Sustainability—use of locally grown foods to minimize transport of food stuffs, use of organic products (which are perceived by many as more sustainable than non-organic products due to more “natural” production methods involving organic soil management, abandonment of pesticides and artificial fertilizers, higher animal welfare, *etc.*) and foods sourced from the wild countryside, shift from meat to plant products and focus on minimizing food waste (by developing a few hundred recipes incorporating reuse) should contribute to reducing environmental impacts associated with food production.

Several studies and reviews [5,6,7,8,9,10] suggest that a healthy diet with high emphasis on nutritious, low-energy components such as fruits, vegetables and seafood tends to be more costly for consumers, and this can pose an important barrier for a healthy lifestyle, especially for consumers in economically and socially deprived households.

Against this backdrop, and using Denmark as an illustrative example, the objective of this study is to investigate the cost-effectiveness of a full-scale implementation of the New Nordic Diet as a health promotion intervention, when taking into account consumer expenditures, as well as environmental and distributional impacts, and hence to indicate, whether promotion of the NND as a strategy to improve public health could be a relevant alternative to other health promotion strategies from a health economic perspective.

## 2. Data and Methods

We apply a combined system of simulation models to assess the long-term cost-effectiveness of a nationwide NND as a health promotion strategy, compared with the current Average Danish Diet (ADD), from a societal perspective. An economic model for consumers’ food expenditure determines the diet composition. This diet composition is then analysed in a consequential life-cycle assessment (LCA) model to determine environmental impacts, which are monetized and included in the cost calculation, and in an epidemiology-based model for food-related non-communicable disease risks to assess public health impacts. The analysis distinguishes between two categories of public health effects: (1) nutrition-related public health effects, which constitute the “effect” componenent in the cost-effectiveness analysis; and (2) environment/pollution-related public health effects, which are incorporated in the cost component of the cost-effectiveness analysis.

### 2.1. Data

The target population of the study is the entire Danish population (the NND diet outlined in Supplement Table S1 for 4–75 year-olds is assumed to be implemented in the entire population). The Consumer Survey 2009–2011 from Statistics Denmark [11] serves as the basis for constructing the ADD scenario. The survey is based on a representative sample of Danish households and shows the size of annual consumption expenditure and its distribution on 55 food and 11 beverage categories for different groupings of households, including five household income classes. According to the survey, an average Danish household in the period 2009–2011 consisted of 1.6 adults and 0.6 children (below 18 years) and had annual food and beverage expenditures of DKK 31,206 (about €4200) and DKK 10,812 (€1450), respectively, including sales taxes but excluding consumption in restaurants, canteens *etc.* All monetary figures are presented in 2010 price level, using an exchange rate of 7.45 between € and DKK [11].

Expenditure data were translated into physical quantity data by dividing the respective cost items by average retail prices per kg. To enable this, the expenditure data were disaggregated as much as possible, using supplementary (and more detailed) 2010-data from GfK Consumer Tracking Scandinavia (www.gfkps.com)—a demographically representative consumer panel containing data from approximately 3000 households, which keep detailed weekly records of their shopping expenditure and volumes. Price estimates for each of these disaggregated commodities were obtained by dividing expenditure by volume of these commodities, and these price estimates were used to translate disaggregated expenditure items from the Consumer Survey into physical quantities, which were then aggregated to a level suitable for the models.

### 2.2. Models

Rational consumers are assumed to adapt to NND in accordance with budget-restricted utility maximization (where utility is derived from the consumption of products, without concern for these products’ impacts on future environment and health prospects). This implies that the resulting consumed quantities of individual commodities within the commodity groups of the NND principles (e.g., mix of different fruits or vegetables) are aligned with the consumers’ preferences as much as possible and that quantities of commodities not mentioned in the NND principles will also be adjusted to yield the highest possible utility to the consumers, while in accordance with the NND principles. Overall, consumed quantities are adjusted such that the marginal utilities of different products are proportional to the perceived prices of these products (which reflect the costs of supplying these products to the consumers).

If consumers are rational utility maximizers, the observed average situation in 2009–2011 (assumed to represent 2010) can be interpreted as the perceived optimal consumption from the average consumers’ points of view, given the prices, incomes, *etc.* at that time (ADD scenario). Hence, this consumption is the solution to the first-order conditions for constrained utility maximization:

(1) $$\begin{array}{c} U_{i}=\lambda \cdot p_{i}\;,i=1,...,N\\ \sum_{i}p_{i}\cdot x_{i}\leq y \end{array}\Rightarrow x^{ADD}\left(p,y\right)$$

Expression (1) states that the marginal utility $U_{i}$ derived from the consumption of commodity i is equal to the price of this commodity $p_{i}$ adjusted for the extent to which the budget constraint y is binding, as represented by the Lagrange multiplier λ. For many consumers, this utility maximization outcome is not consistent with the NND principles.

In order to estimate a preference-based diet fulfilling the NND principles, we introduce additional dietary constraints to the utility maximization problem:

- Unchanged total energy intake (coefficient $e_{i}$ represents energy content in commodity $x_{i}$)
- maximum limits $S_{j}$ to intake of some foods or nutrients (coefficient $s_{ji}$ characterizes commodity $x_{i}$ with respect to constraint j) , and
- minimum limits $Q_{h}$ to intake of some other foods or nutrients (coefficient $q_{hi}$ characterizes commodity $x_{i}$ with respect to constraint h)

The three types of constraints have associated Lagrange multipliers (ψ, $\eta_{j}\geq 0,\theta_{h}\leq 0$) representing the degree to which they are perceived binding for the consumers’ choices. Hence, the NND consumption is the solution to the augmented first-order conditions for constrained utility maximization:

(2) $$\begin{array}{l} \begin{array}{c} U_{i}=\lambda \cdot p_{i}\;+\psi \cdot {\text{e}}_{\text{i}}\cdot x_{i}+\sum_{\text{j}}\eta_{\text{j}}\cdot s_{ji}+\sum_{\text{h}}\theta_{\text{h}}\cdot q_{hi}\;,i=1,...,N\\ \begin{array}{l} \sum_{i}p_{i}\cdot x_{i}\leq y\\ {\sum_{i}e}_{i}\cdot x_{i}^{NND}={\sum_{i}e}_{i}\cdot x_{i}^{ADD} \end{array}\\ \sum_{i}s_{ji}\cdot x_{i}\leq S_{j}\\ \sum_{i}q_{hi}\cdot x_{i}\geq Q_{h} \end{array}\\ \Rightarrow x^{NND}\left(p+\frac{\psi }{\lambda }\cdot e+\sum_{j}\frac{\eta_{\text{j}}}{\lambda }\cdot s_{j}+\sum_{h}\frac{\theta_{\text{h}}}{\lambda }\cdot q_{h},y\right)=x^{NND}\left(\tilde{p},y\right) \end{array}$$

In this framework, an imposition of the New Nordic Diet alters the perceived relative (implicit) prices (including impacts of the Lagrange multipliers) of different food and beverage commodities. Compared with market prices, the relative implicit prices of commodities to be promoted will be lower (because $\theta_{h}\leq 0$), and implicit prices of commodities containing substances to be reduced will be higher (because $\eta_{j}\geq 0$).

The shape of the hence derived demand functions depends on the shape of the marginal utility function $U_{i},i\in \left\{1,...,N\right\}$. Normally, $U_{i}$ is expected to be a convexly decreasing function of $x_{i}$, whereas the relationship between $U_{i}$ and the consumed quantity of another good $x_{g}$ depends on substitutability or complementarity between goods i and g. These relationships are reflected in own- and cross-price elasticities of consumers’ demands for these products. The economic model consists of a 97 × 97 matrix of such price elasticities for all combinations of food and beverage prices and quantities, which was estimated on the basis of household-level panel data from GfK ConsumerTracking Scandinavia for five income categories of households, corresponding to the income groups in Statistics Denmark’s Consumer Survey.

Given own- and cross-price elasticities for the demand of the respective commodities, $\varepsilon_{ji}$, the vectors of Lagrange multiplier ratios (ψ/λ, η/λ, θ/λ) can be determined by solving the equations:

(3) $$\begin{array}{l} \sum_{i}e_{i}\cdot x_{i0}\cdot \left(1+\sum_{g}\varepsilon_{gi}\cdot \frac{e_{g}\cdot \psi +\sum_{v}s_{vg}\cdot \eta_{v}+\sum_{w}q_{wg}\cdot \theta_{w}}{\lambda \cdot p_{g}}\right)=\sum_{i}e_{i}\cdot x_{i0}\\ \sum_{i}s_{ji}\cdot x_{i0}\cdot \left(1+\sum_{g}\varepsilon_{gi}\cdot \frac{e_{g}\cdot \psi +\sum_{v}s_{vg}\cdot \eta_{v}+\sum_{w}q_{wg}\cdot \theta_{w}}{\lambda \cdot p_{g}}\right)=S_{j}\\ \sum_{i}q_{hi}\cdot x_{i0}\cdot \left(1+\sum_{g}\varepsilon_{gi}\cdot \frac{e_{g}\cdot \psi +\sum_{v}s_{vg}\cdot \eta_{v}+\sum_{w}q_{wg}\cdot \theta_{w}}{\lambda \cdot p_{g}}\right)=Q_{h} \end{array}$$

For example, if the maximum recommended intake of added sugar (an element in S) is lower than the actually observed intake, the Lagrange multiplier of this restriction will be positive, and hence the implicit prices of commodities containing added sugar will be higher than the observed market prices—providing an implicit incentive to consume less of these commodities—and vice versa for goods, which should be promoted according to the NND principles. Having determined the Lagrange multipliers, we can calculate the implicit prices of all 97 commodities in the model:

(4) $${\tilde{p}}_{i}=p_{i}+\psi /\lambda \cdot e_{i}+\sum_{v}\eta_{v}/\lambda \cdot s_{vi}+\sum_{w}\theta_{w}/\lambda \cdot q_{wi}\;,i\in \left\{1,...,N\right\}$$

These implicit prices would give the consumer the incentive to choose a combination of foods and beverages adhering to the NND principles. Using the estimated price elasticities, we can calculate the consumed quantities in the NND:

(5) $${\tilde{x}}_{i}=x_{i0}\cdot \left(1+\sum_{g}\varepsilon_{gi}\cdot \left(\frac{{\tilde{p}}_{g}-p_{g}}{p_{g}}\right)\right)\;,i\in \left\{1,...,N\right\}$$

From the ambitions of Nordic gastronomic identity and environmental sustainability the NND principles suggested that ≥75% of the food should be organic and 95% of Nordic origin [4]. Based on the GfK dataset, we have estimated average prices for both organic and non-organic varieties of each commodity, where possible. In order to determine those goods that would most likely be organic if consumers are economically rational, we calculated price differentials (per calorie) between organic and non-organic varieties. These price differentials ranged from 10–20 per cent for some vegetables (e.g., peas, frozen/processed vegetables), dairy and grain products, to 50–100 per cent for some other vegetables (e.g., cabbage, cucumbers, onions), fruits and meat products. We assumed that current choices of organic varieties for each commodity would be maintained (as a minimum), and that additional choice of organic varieties would occur from the low end of price differentials, until 75% of total calories were reached.

The available data material did not allow distinction between goods of Nordic *versus* non-Nordic origin. For many livestock products, large shares of the domestic consumption are of domestic origin and for those products, no significant price differentials were assumed. In contrast, imports constitute considerable shares of domestic fruit and vegetable consumption, and restrictions to only include Nordic produced fruits and vegetables would imply higher prices, although the NND principle of utilizing seasonal variation in supply will tend to reduce this price differential. In the calculations, we assumed average price differentials of 10% for fruits and vegetables, which are supplied from both imports and domestic production (e.g., apples, pears, plums, strawberries, tomatoes, cucumbers, bell peppers, cabbage), whereas no price differential was assumed for goods with a currently high domestic share (e.g., roots, onions, potatoes).

The price differentials between organic and non-organic, and between Nordic and non-Nordic, varieties were used to obtain adjusted prices ${\hat{p}}_{i}$ that reflect these price differentials. These adjusted prices were used to calculate the households’ NND food budget as: $\sum_{i}{\hat{p}}_{i}\cdot {\tilde{x}}_{i}$ for each of the five income groups.

*Environmental impacts* of the ADD and the NND were evaluated for 15 impact categories (human toxicity from carcinogens and non-carcinogens, respiratory organics and inorganics, ionizing radiation, ozone layer depletion, aquatic and terrestrial ecotoxicity, aquatic and terrestrial eutrophication, nature occupation, global warming, acidification, photochemical ozone effect on vegetation, and non-renewable energy, cf. Supplement Table S2), based on consequential life cycle assessment (cLCA) using the Simapro 8 software with “kg food or beverage” as the functional unit, and the international Ecoinvent 3 [12] and the Danish LCA food databases [13], supplemented with adequate data from the literature where needed.

The life cycle assessment (LCA) model focused on the environmental characteristics of different commodities, distinguishing between domestic and imported goods. Commodities were aggregated into composites based on their environmental characteristics [14]. Because the aggregation of the LCA model deviated from the COICOP aggregation used in Statistics Denmark’s data, linkages between the COICOP classification and the LCA model classification were established using the more disaggregated GfK data material, thus enabling a translation of results from the economic simulation to the LCA framework to assess environmental impacts of the dietary differences between NND and ADD.

In order to aggregate environmental impacts and budgetary impacts for the consumers, we attempt to monetize the environmental effects, although it is recognized that such monetization may be considered as controversial, and that the results of the monetization may depend on the methodology used. Different approaches to monetization exist, including: (1) Cost-of-illness/cost-of-damage approaches, evaluated on the basis of productivity effect estimates or on preference measurement [15]; or (2) Abatement cost approaches (e.g., [16]). Due to the controversial nature of the subject, we apply two alternative monetizations, both based on the Stepwise method [17,18], where a cost-of-illness/cost-of-damage productivity loss approach has been used (Supplement Table S2). One monetization is taken from Weidema [18], who—based on the literature—translated environmental effects with human health impacts into loss of quality adjusted life years (QALY). Assuming that a QALY represents the loss of one person-year productivity, the value of one QALY was estimated as the maximum annual earning potential (estimated as the US GDP/capita—equivalent to €74,000 in 2003-price level [18] or €77,000 in 2010-price level—adjusted upwards for long-run removal of various imperfections in the US economy) from a global perspective—as several of the environmental impacts are of a global nature. Effects on ecology and biodiversity were measured in biodiversity-adjusted hectare years (BAHY), assuming that the value of one BAHY is equivalent to the value of 0.02 QALY, based on previous work by Weidema [18]. In the alternative monetization, we have used the unadjusted US GDP/capita as a measure for the value of one QALY (with derived consequences for the value of one BAHY). Other environmental valuation principles are used in the literature, but most of the estimates lie within the ranges used here [15].

An impact fraction model was developed and linked to the difference in food and beverage consumption from ADD to NND to assess potential *health risk effects* of this dietary shift. Assuming that a dietary behavior (e.g., fruit and vegetable consumption below a certain threshold) can be considered as high-risk, the relative risk (RR) represents the ratio of a certain health risk (e.g., for death due to stroke) for below- and above-threshold consumption, respectively. If $\alpha_{b}$ and $\alpha_{a}$ represent the shares of individuals exposed to the considered risk factor before and after the change, respectively, the impact fraction (IF) can be calculated as [19]:

(6) $$IF_{d}=\frac{\left(\alpha_{b}-\alpha_{a}\right)+RR\cdot \left(\alpha_{a}-\alpha_{b}\right)}{\left(1-\alpha_{b}\right)+RR\cdot \alpha_{b}}\;$$

This impact fraction represents the relative change in aggregate health risk (or disease burden) for disease d following from a change in risk factor exposure, given by the difference: $\alpha_{a}-\alpha_{b}$.

We focus on the impacts of 10 dietary factors on the risks of up to 6 diet-related non-communicable diseases (cardiovascular disease, stroke, diabetes, stomach cancer, lung cancer and breast cancer). A systematic search for literature on associations between these dietary factors and health risks was conducted, and the findings were synthesized into relative risk figures [20,21,22,23,24,25,26,27,28,29,30,31,32,33,34,35,36,37,38,39,40,41,42,43,44,45,46,47,48,49,50,51,52,53] (Supplement Table S3).

A shift from ADD to NND will tend to increase the average intake of fruits and vegetables. This will (from a partial perspective) reduce the risk of all the considered chronic diseases—represented by RR-parameters below 1. Hence, most of the principles within the NND seem to imply lower health risks (which is also one of the main objectives of the NND). Despite an *a priori* presumption that sugar would also constitute a risk factor for some of these diseases, no studies with RR estimates for sugar (except for sugared drinks) were found. Nevertheless, if the *a priori* presumption holds, we may under-estimate the health benefit from a dietary change towards NND.

Using Statistics Denmark’s Consumer Survey and results from the National Dietary Survey [54], we estimate normal distributions of the above 10 dietary components in the Danish adult population (assuming that the same relative variation holds for children), and hence the shares of risk-exposed individuals in the ADD, $\alpha_{b}$, to represent the heterogeneity in households’ food consumption patterns. Assuming that the NND intervention will modify the mean values of these distributions—leaving the standard deviations and shape of the distributions unaffected—we then calculate the corresponding shares of risk-exposed individuals in the NND, $\alpha_{a}$, and are thus able to calculate the impact fraction for each combination of risk factor and health risk using expression (6). We translate these impact fractions into absolute health effect ΔH using the expression:

(7) $$\Delta H=\sum_{d}IF_{d}\cdot \frac{DALY_{d}^{EUR_{a},2004}}{deaths_{d}^{EUR_{a},2004}}\cdot deaths_{d}^{DK,2010}$$

where disease-specific impact fractions ($IF_{d}$) are multiplied by estimates of the disease burden (DALY) in Denmark for the respective health risks, obtained by ratios between the most recent (2004) WHO-figures on DALY and number of deaths in Western Europe ($EUR_{a}$) [55], which are multiplied by number of deaths from the respective diseases in Denmark in 2010, where the ratio between DALY and number of deaths for the respective diseases was assumed to have been stable from 2004 to 2010. If the introduction of NND would also narrow the distributions of dietary patterns, the potential positive health impacts would presumably be larger than those presented in the calculations below. Hence, we have annual figures for the costs (net of environmental benefits) and the health effects of the NND, compared to the ADD. As the health effects of dietary change are likely to occur with several years’ time lag, we have discounted the health effects by 10 years in the calculation of cost-effect ratios. Following recommendations from several authors in health economics (e.g., [56]), we have used a relatively low discount rate for these health effects, *i.e.*, an annual discount rate of 1%.

## 3. Results

Solving the utility maximization problem for the NND (2) yields implicit prices associated with constraints for dietary components such as fruits (€0.18–0.41 per kg lower than the average market price), seafood (€0.25–1.20 per kg below average market price), meat (€0–0.63 per kg above average market price), saturated fat (upward price adjustment of €0.63–2.42 per kg saturated fat), *etc.*, where the intervals represent variation across the five income groups. These implicit prices represent the price structure that would give the average consumers within the five income groups the economic incentive to consume a diet consistent with the NND principles. The overall composition of the national food budget in the ADD and the NND is shown in Table 1. 

**Table 1:** Aggregate consumption effects, 1000 tonnes and million €.

	Consumed Quantities (1000 tonnes)		Expenditure, mill. €	
	ADD	NND	ADD	NND
Grain-based foods	1005	1317	1789	1857
Meat	293	179	2722	1670
Seafood	65	81	603	745
Dairy and fats	960	771	1898	1646
Fruits and vegetables	847	1432	2321	5185
Other foods	357	305	1614	1718
Foods total			10,948	12,821
Beverages	1204	1304	2644	2898
Total			13,591	15,719

The NND consumption of grain-based foods, seafood, fruits and vegetables is higher than in the ADD scenario (with an increase in whole grain consumption constituting the difference for grain based foods), whereas the consumption of meat, dairy products, fats and other foods is lower. Although consumption of grain-based foods is higher, the expenditure is not higher, because the consumption is more on grains, flour *etc.* and less on industrially produced bread and pasta.

Overall, the NND consumer expenditure for food and beverages is about €1.87 billion and €0.25 billion higher, respectively, totaling an increase in food and beverage expenditure of about 16%. Out of this, the use of 75% organic products accounts for about €0.9 billion, and the requirement of 95% products of Nordic origin contributes by €0.4 billion. The expenditure for fruits and vegetables shows the largest increase, but also the expenditures for seafood and grain-based foods increase, whereas the expenditures for meat and dairy products decrease. In addition to the effects of increased organic and Nordic-origin shares within consumption, the effects also represent changes in consumed quantities, as represented by the above-mentioned system of price elasticities. Within fruits and vegetables, an increase in consumption of fruits is determined in the NND (in line with the NND-principles), but the largest increase takes place for bananas and citrus fruits (which cannot be produced in the Nordic countries), because of relatively strong consumer preferences for these fruit types. Hence, the principle of 95% Nordic origin is satisfied in other product categories such as meat, dairy, vegetables and grain-based commodities.

We can estimate the consequences of the changes on different environmental indicators using the above-mentioned cLCA framework. Measured in monetary values, five environmental indicators stand out: Nature occupation, global warming, respiratory inorganics, non-carcinogenic human toxicity and terrestrial eutrophication. Together, these five indicators account for about 97% of the monetized value of the 15 environmental indicators, with nature occupation, global warming and respiratory inorganics as the most significant, in the ADD baseline.

**Figure 1:**
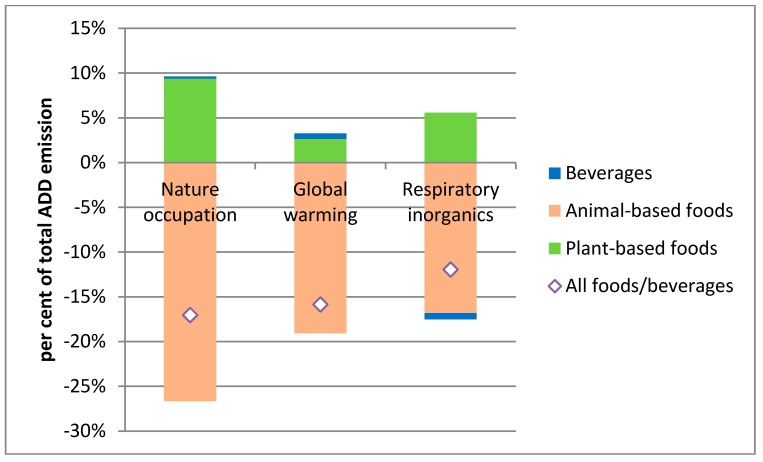
Effects of NND on most important environmental externalities.

Figure 1 illustrates the relative changes in the three most important environmental indicators, decomposed into contributions from changes in consumption of beverages and animal- and plant-based foods. For all three indicators, the NND has more favorable environmental outcomes than the ADD: 17% lower for nature occupation, 16% for global warming, and 12% for respiratory inorganics. These environmental improvements stem primarily from the reduced consumption of animal-based products, whereas increased consumption of plant-based products reduces this effect. Non-carcinogenic human toxicity is however significantly reduced (74%) due to the high share of organic products in the NND.

Depending on the method of monetization, the monetary value of the environmental improvements was estimated to be €0.5–0.9 billion, which could be deducted from the above-calculated food expenditure increase to obtain a societal net figure for the cost increase.

**Figure 2:**
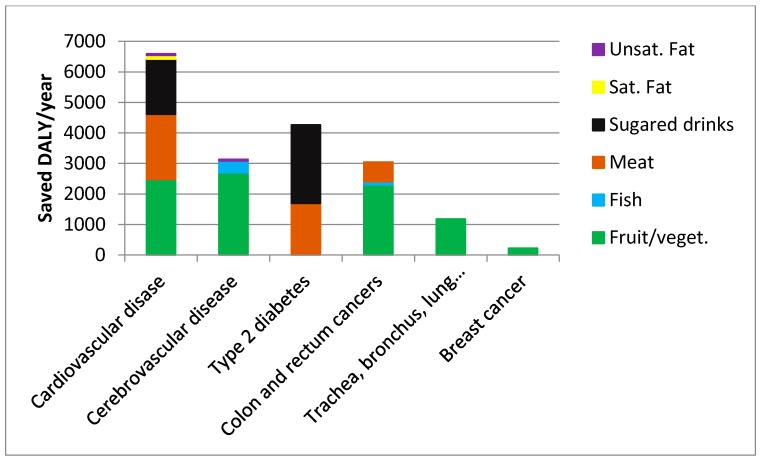
Saved DALY per annum in Denmark from selected diet-related health problems.

Based on the impact fraction methodology described above, we have calculated the effects of the dietary change (from ADD to NND) on the disease burden from the 6 mentioned chronic diseases (Figure 2). For example, the dietary change is calculated to save about 6500 DALY/year from cardiovascular disease, with main contributions from increased consumption of fruit and vegetables, reduced consumption of meat and reduced consumption of sugared drinks, but minor effect from changed consumption of fats. The dietary change is also estimated to have a marked effect on disease burdens from stroke and diabetes, whereas about 3000 and 1200 DALY/year are calculated to be saved from colorectal cancer and lung cancer, respectively. In total, an estimated 18000 DALY will be saved per year in Denmark from these six chronic diseases.

Combining the aggregate consumption expenditure, monetized environmental benefits and discounted calculated health outcomes, the cost-effectiveness of the New Nordic Diet as a public health promotion tool was calculated. Using the monetization of environmental effects by Weidema [18], we obtain (€2.1 bill. − €0.9 bill.)/18396 DALYs ~ €73,000/DALY, whereas the alternative monetization yields €94,000/DALY.

Having modelled five income classes of households enables assessing some distributional effects of the dietary shift (Table 2). For ease of comparison between households with different compositions, economic figures are measured on a per adult-equivalent basis (one child is assumed equivalent to 0.7 adults). On average, the additional budgetary spending on food and beverages is €417 per adult-equivalent, corresponding to about 16% of the ADD spending on food and beverages. In absolute terms, this food and beverage budget increase is similar across income groups, and is hence relatively larger for the lower-income groups (16.2%–17.2% for low-income households *versus* 15.2%–15.7% for high-income households).

**Table 2:** Distributional effects, €/adult-equivalent/year.

	ADD	NND	Environmental Benefit	Net Cost/DALY	Food and Beverage Expenditure	Disease Reduction (DALY)
	€/adult_Equivalent/year		€/Adult Equivalent	€1.000	per cent Difference	per cent Reduction
I	2323	2699	110–179	89–121	16.2	10.1
II	2383	2794	110–179	69–89	17.2	15.0
III	2482	2860	110–179	62–84	15.2	14.1
IV	2715	3143	110–179	70–90	15.7	14.4
V	2813	3240	110–179	78–99	15.2	13.4
All income classes	2667	3084	110–179	73–94	15.7	13.8

Note: Income classes—I: < DKK 150,000 (<€20,000), II: DKK 150–299,999 (€20–40,000), III: DKK 300–499,999 (€40–67,000), IV: DKK 500–799,999 (€67–108,000), V: > DKK 800,000 (>€108,000).

Counterbalancing the value of environmental benefits (€110–179 per adult-equivalent per year—assumed equally distributed across income groups) reduces the net cost to a range between €238 and €307 per adult-equivalent. The health effects displayed in Figure 2 translate into an average reduction in aggregate disease burden from the six diseases by 13.8%, which is distributed across income groups according to their relative health risks estimated from current (ADD) dietary patterns. Except for the lowest income group, the health promoting effect of the dietary shift tends to be decreasing with income level, as measured by percentage reduction in the number of DALY. And taking into account that the disease burden is relatively low in high-income groups for most of the considered disases [57], this decreasing trend in health promoting effect with income level prevails when the health effect is measured in absolute terms. The relatively low health effect in the lowest income group is explained by diets that are somewhat healthier in this income group (including lower intake of meat, fat and sugared drinks) and some of the responses to the ADD-NND shift are smaller (e.g., the consumption of sugared beverages). Combining these figures to consider the cost-effectiveness of NND as an intervention to reduce disease burden from the six diseases, we find a net cost of €73,000–94,000 per saved DALY (depending on the valuation of environmental benefits). This cost-effectiveness measure shows some variation across income groups, with the most favorable cost-effectiveness ratios for medium-income households, and the least favorable ratio for households with relatively high or low incomes.

## 4. Discussion

The study combines simulation models from different subject areas into a model system which is used for evaluating the cost-effectiveness of a large-scale transition from the current dietary pattern to a healthy and sustainable regional diet (New Nordic Diet) as a health promotion instrument at population level. The combination of models represents a relatively unique approach to such cost-effectiveness analyses, which yields the opportunity of taking both direct and external net costs into consideration. We find that a full-scale implementation of the New Nordic Diet in Denmark might reduce the disease burden from cardiovascular diseases, diabetes and four cancer types by more than 18,000 DALY per year in Denmark, compared with the current average Danish diet, with a cost-effect ratio of €73,000–94,000 per DALY, when account of environmental benefits is taken (and almost €128,000 per DALY if these environmental benefits are disregarded). The NND assumes that organic products constitute at least 75% of the diet, and this attribute contributes substantially to the costs (but is not assumed to contribute to the public health effects in terms of the considered diseases, as no strong evidence was found to support such effects). If this requirement were disregarded (as a sensitivity analysis), some of the environmental benefits (including nature occupation and respiratory inorganics) would be slightly higher and some (e.g., global warming) would be lower, and the cost-effect ratio reduced from €128,000 to €74,000 per DALY (ignoring environmental benefits). The finding that some environmental benefits would be higher without the organic requirement is due to the lower intensity (yield per hectare or per animal-year), implying that emissions that are related to hectares or animals will come out higher when measured per output unit in organic farming—even though emissions per hectare or per animal may be lower for organic than for conventional production. And if the additional requirement that 95% of the diet should be of Nordic origin were also disregarded, this cost-effect ratio would be reduced to about €50,000 per DALY, which is within the size of the monetized environmental benefits—depending on the principles for monetizing these benefits.

These figures may be compared with estimated costs, effects and cost-effectiveness properties of other community- or population-based health promotion interventions in the literature. Several other studies have found comparable costs for healthy diets compared to less healthy diets [8,9,58,59]. Trichopolou *et al.* [2] found a significantly lower mortality for individuals adhering to the Mediterranean diet. Hence, one may expect positive cost-effect ratios, as we found in the present study. However, studies with comparable interventions and outcome measures are relatively hard to find in the literature. Many studies address health promoting dietary interventions targeted towards high-risk individuals (e.g., [60,61,62]) and show better cost-effectiveness properties (which should also be expected, because the potential average health effects are relatively larger). Other studies may be population-based but use more narrow cost concepts (e.g., direct implementation costs for health promotion or health care sectors, but ignoring dietary costs for consumers) than ours, also showing relatively high effectiveness per cost unit [63,64,65]. WHO recommendations suggest interventions with a cost in the range from one to three times GDP *per capita* to be considered as cost-effective [66]. With a Danish GDP per capita of €43,500 in 2010, this suggests a threshold of about €130,000 per DALY, which might indicate that the NND intervention can be considered as cost-effective, especially when environmental impacts are taken into account. When comparing the findings of the present study with those of other health promotion studies, it should be kept in mind that the NND is not purely a health promoting intervention, but also carries broader visions in fields such as gastronomic identity, lifestyle, familiarity with nature’s resources *etc.*, which could be presumed to add further—but non-quantifiable—value to the New Nordic Diet, as compared with other health promotion interventions.

We have assumed that consumers ignore environmental and future health impacts of the consumption in their decision making. If parts of these impacts are internalized in their baseline consumption decision, this may introduce uncertainty to our cost-effectiveness calculations. Our cost estimates do not include any implementation or transaction costs in connection with the considered dietary change (e.g., promotion activities, preparation of recipes, *etc.*). The NND implies significant changes in dietary habits for a considerable share of the population, and this raises a number of issues related to adaptability or acceptability [67]. Such issues may have implications for the transaction costs associated with implementing such dietary changes. Hence, the presented figures may under-estimate the total costs of such a major change.

In order to ease consumers’ adoption of the NND, substantial effort has been devoted to the development of easy-to-apply recipes and to the popular dissemination of these recipes in top-selling recipe books, in magazines, in electronic media, *etc.*—efforts that have been able to benefit from recent years above-mentioned international success of the New Nordic Cuisine in Danish gourmet restaurants and the publicity around this success [3]. Nevertheless, the scenario of a full-scale implementation of the NND is still considered to face serious challenges. One particular challenge in relation to the NND principle of at least 95% of the ingredients being of Nordic origin is the relatively low availability of some products (especially within fruits and vegetables) during a period of the year. The recipe material addresses this challenge by taking into account the seasonality in the availability of Nordic-origin food products. Furthermore, as noted in the results section, the modelled NND includes some consumption of imported fruits, which may compensate for low availability of local fruits in the winter and spring seasons.

In the modelling of NND’s health effects, we assumed shifts in mean values of statistical distributions of different dietary intakes (fruits, vegetables, seafood, *etc.*), while keeping the standard deviations and correlations of the individual intake components unchanged, due to lack of estimates of these parameters. This assumption introduces uncertainty to the calculated health effects. For example, the study by Jensen and Poulsen [57] suggested a larger variation in calculated food expenditure within the NND than the ADD, but the reverse pattern could also be imagined, and intake distributions may have different skewness properties and correlation patterns in the two diets. Moreover, it should be noted that the quantitative health impact assessment only includes six diseases, suggesting that these health impact estimates should be considered as lower-end estimates of the total health benefits. Furthermore, the assumption of a constant relationship between DALY and number of deaths for these six diseases from 2004 to 2010 also adds uncertainty to the health impact estimates.

In the food expenditure calculation, we have assumed that current market prices will prevail after a dietary switch, and hence that there are no constraints on the availability and accessibility of the ingredients in the NND. A full-scale switch may however impose radical changes in the demand-supply balance on the markets for some products within the NND (e.g., fresh fruits, berries, vegetables, seafood and wild herbs) as well as in the ADD (e.g., meat or processed foods). For internationally traded commodities, this is not supposed to affect prices significantly, but for commodities that are traded in more isolated (e.g., local) markets, a larger demand may lead to a price increase, whereas lower demand can lead to reduced prices. As the NND has high emphasis on locally produced, fresh and organic products traded in relatively small markets, the shift may imply an upward price push of these commodities—at least in the short-to-medium term, suggesting that the estimated increase in consumer expenditure may represent a lower-end estimate in the short run, but may be realistic in a long-run perspective.

The aim of this study has been to assess the cost-effectiveness properties of a full-scale implementation of a New Nordic Diet in Denmark and thereby estimate the full potential of a change in diet composition, rather than to guide an implementation of the diet. Hence, in the analyses, the New Nordic Diet scenario is assumed implemented without considering potential policy vehicles to obtain this dietary change. The economic model analysis yielded implicit prices in the NND and their deviations from current market prices, and these deviations provide some indication of the incentive barriers that should be overcome in order to realize the NND scenario. Some of these barriers could be changed by modifying food prices via taxes or subsidies, although Denmark’s membership of the European Union *etc.* may pose limits to the extent to which consumers’ access to imported food commodities can be governed by such public regulation tools. Other ways to reduce the barriers for extending the NND to broader groups could be an improved performance of the markets for these foods and/or increased retailer focus on NND components in their marketing and promotion activities, but changes in consumers’ preferences will probably also be necessary, if the NND scenario should become reality.

The present study is based on a combination of empirically based simulation models from different scientific disciplines. Naturally, this combined analysis inherits existing weaknesses from the different components of the model framework, including e.g., uncertainty on the parameters of the respective model tools (such as price elasticities, RR estimates, environmental impact coefficients and monetization of the environmental impacts). The model approach describes changes for “average” households within different income groups but disregards the heterogeneity in e.g., food consumption patterns within these income groups, and this may add some uncertainty to the quantitative results—not least with regard to the estimation of health impacts. Despite such weaknesses and uncertainties, the above analysis is considered to yield new and important quantitative insights into the potential health economic prospects of major dietary changes, such as a nation-wide implementation of the New Nordic Diet.

## 5. Conclusions

The present study finds that on an aggregate level, the New Nordic Diet is on average about 16% more expensive for the consumers than the current Average Danish Diet at the current market prices. The cost difference is relatively larger in low-income households, which is in line with results of previous cost studies comparing healthy with less healthy diets. However, taking into account positive environmental effects (which could be considered as a societal benefit), the cost increment is reduced to 9%–12%. The shift is found to reduce the aggregate disease burden of six chronic diseases (cardiovascular disease, stroke, diabetes mellitus, colorectal cancer, breast cancer and lung cancer) by about 14%, yielding a calculated cost-effectiveness ratio of about €73,000–94,000 per DALY saved. As both health impacts and cost differences tend to be relatively larger in low-income households, there is no clear socio-economic pattern in the cost-effectiveness properties. If the assumptions of 75% organic products and 95% products of Nordic origin are relaxed, the cost-effect ratios would be substantially lower. Hence, implementation of the New Nordic Diet could be an economically relevant strategy for promotion of public health, provided moderate transaction costs associated with such implementation.

## Supplementary Materials

**Table S1:** Overview of the average daily content of the dietary components in the New Nordic Diet (NND) in relation to the average daily content in the Average Danish Diet (ADD) (energy-adjusted intake per 10 MJ of all persons aged 4–75 years).

Dietary Component	Average Content in the NND (g/day)	Average Content in the Danish Population’s Diet 2010 (g/day)
**Ingredients, g/10 MJ**		
Fruit	>300 *(250*–*350)*	240
Vegetables	>400 *(350*–*450)*	181
Including		
- berries	*(50*–*100)*	5
- cabbages	>29 *(25*–*35)*	9
- root vegetables	*>150*	38
- legumes	*>30*	7
Fresh herbs	As much as possible *(**≥1**)*	
Potatoes	>140 *(140*–*160)*	106
Plants and mushrooms from the wild countryside	5 *(3*–*7)*	<1
Whole grains	*>75*	36
Nuts	*>30*	1
Fish and shellfish	>43 *(40*–*50)*	22
Seaweed	5 *(3*–*7)*	<1
Free-range livestock	85–100 *(90*–*110)*	143
Including		
- game	>4 *(2*–*6)*	<1
**Macronutrients *etc.***		
Protein (E%)	18 *(15*–*23)*	15
Total carbohydrate (incl. fibres), E%	52 *(48*–*56)*	50
Added sugar (E%)	*<10*	
Total fat (E%)	30 *(25*–*35)*	35
Saturated fat (E%)	*<10*	15
Nordic produce (%)	*≥95*	
Organic (%)	*≥75*	

Italics: Limits implied by the NND. E%: Energy per cent. Source: [4].

**Table S2:** Monetized environmental effects (2010 price level).

	Unit	COI/COD Approach, Based on Adjusted US GDP/Capita	COI/COD Approach, Based on Unadjusted US GDP/Capita
Human toxicity, carcinogens	€/kg C_2_H_3_Cl-eq	0.2823	0.2854
Human toxicity, non-carcinogens	€/kg C_2_H_3_Cl-eq	0.2826	0.2854
Respiratory inorganics	€/kg PM_2.5_-eq	70.4198	45.4682
Ozone layer depletion	€/kg CFC-11-eq	106.0406	70.2595
Ecotoxicity, aquatic	€/kg TEG-eq w	0.0000	0.0000
Ecotoxicity, terrestrial	€/kg TEG-eq w	0.0012	0.0006
Nature occupation	€/m^2^ agr.land	0.1293	0.0690
Global warming	€/kg CO_2_-eq	0.0865	0.0459
Acidification	€/m^2^ UES	0.0081	0.0043
Eutrophication, aquatic	€/kg NO_3_-eq	0.1056	0.0564
Eutrophication, terrestrial	€/m^2^ UES	0.0130	0.0070
Respiratory organics	€/pers × ppm × h	0.2662	0.1705
Photochemical ozone vegetation	€/m^2^ × ppm × hours	0.0004	0.0003

Source: [17]; ionizing radiation and non-renewable energy were not monetized.

**Table S3:** Relative diet-related health risks.

	Cardio-Vascular Disease	Stroke	Diabetes	Stomach Cancer	Lung Cancer	Breast Cancer
Fruits and vegetables exposure, 200 g/day	1	1	1	1	1	1
300 g/day	0.97	0.95	1	0.77	0.89	0.95
400 g/day	0.93	0.89	1	0.77	0.89	0.95
500 g/day	0.88	0.82	1	0.77	0.89	0.95
600 g/day	0.83	0.74	1	0.77	0.89	0.95
Fish, exposure, 10 g/day	1	0.93	1	0.87	1	1
20 g/day	1	0.78	1	0.89	1	1
50 g/day	1	0.73	1	0.83	1	1
150 g/day	1	0.48	1	0.84	1	1
Red meat exposure +100 g/day	1.00	1	1.18	1.08	1	1
Processed meat exposure +50 g/day	1.42	1	1.3	1.01	1	1
Sugared drinks exposure, 10 g/day	1	1	1	1	1	1
25 g/day	1	1	1.06	1	1	1
160 g/day	1.04	1	1.5	1	1	1
250 g/day	1.23	1	1.85	1	1	1
Whole grain exposure +10 g/day	0.90	1	0.79	1	1	1
Total fat exposure, +5 E%	1.00	1	1	1	1	1
Saturated fat exposure, +5 E%	1.05	1	1	1	1	1
Monounsaturated fat exposure, +5 E%	0.96	0.91	1	1	1	1
Polyunsaturated fat exposure +5 E%	0.92	0.91	1	1	1	1

Sources: [20,21,22,23,24,25,26,27,28,29,30,31,32,33,34,35,36,37,38,39,40,41,42,43,44,45,46,47,48,49,50,51,52,53].

## List of Abbreviations

OPUS: Acronym for the project “Optimal well-being, development and health for Danish children through a healthy New Nordic Diet”
NND: New Nordic Diet
ADD: New Nordic Diet
